# Implementation of human biomonitoring in the Dehcho region of the Northwest Territories, Canada (2016–2017)

**DOI:** 10.1186/s13690-018-0318-9

**Published:** 2018-12-03

**Authors:** Mylène Ratelle, Kelly Skinner, Matthew J. Laird, Shannon Majowicz, Danielle Brandow, Sara Packull-McCormick, Michèle Bouchard, Denis Dieme, Ken D. Stark, Juan Jose Aristizabal Henao, Rhona Hanning, Brian D. Laird

**Affiliations:** 1 School of Public Health and Health Systems, Faculty of Applied Health Sciences, University of Waterloo, 200 University Ave W, Waterloo, ON Canada; 2 Faculty of Medicine, Université de Montréal, 2900 Edouard-Montpetit, Montreal, QC Canada

**Keywords:** Dene, First nations, Biomonitoring, Metals, Traditional foods, North, Biomarker, Exposure, Risk assessment

## Abstract

**Background:**

Human biomonitoring represents an important tool for health risk assessment, supporting the characterization of contaminant exposure and nutrient status. In communities where country foods (locally harvested foods: land animals, fish, birds, plants) are integrated in the daily diet, as is the case in remote northern regions where food security is a challenge, such foods can potentially be a significant route of contaminant exposure. To assess this issue, a biomonitoring project was implemented among Dene/Métis communities of the Dehcho region of the Northwest Territories, Canada.

**Methods:**

Participants completed dietary surveys (i.e., a food frequency questionnaire and 24-h recall) to estimate food consumption patterns as well as a Health Messages Survey to evaluate the awareness and perception of contaminants and consumption notices. Biological sampling of hair, urine and blood was conducted. Toxic metals (e.g., mercury, lead, cadmium), essential metals (e.g., copper, nickel, zinc), fatty acids, and persistent organic pollutants (POPs) were measured in samples.

**Results:**

The levels of contaminants in blood, hair and urine for the majority of participants were below the available guidance values for mercury, cadmium, lead and uranium. However, from the 279 participants, approximately 2% were invited to provide follow up samples, mainly for elevated mercury level. Also, at the population level, blood lead (GM: 11 μg/L) and blood cadmium (GM: 0.53 μg/L) were slightly above the Canadian Health Measures Survey data. Therefore, although country foods occasionally contain elevated levels of particular contaminants, human exposures to these metals remained similar to those seen in the Canadian general population. In addition, dietary data showed the importance and diversity of country foods across participating communities, with the consumption of an average of 5.1% of total calories from wild-harvested country foods.

**Conclusion:**

This project completed in the Mackenzie Valley of the Northwest Territories fills a data gap across other biomonitoring studies in Canada as it integrates community results, will support stakeholders in the development of public health strategies, and will inform environmental health issue prioritization.

**Electronic supplementary material:**

The online version of this article (10.1186/s13690-018-0318-9) contains supplementary material, which is available to authorized users.

## Introduction

Exposure characterizations are a critical component of human health risk assessments, providing key insights on the environmental determinants of health at the local, regional, national, and global scales. Contaminant exposure is spatially and temporally dependent and can be influenced by natural and anthropogenic factors that impact air and water quality as well as the integrity of locally-harvested food sources. In remote subarctic Indigenous (First Nations, Métis and Inuit) communities of the Northwest Territories (NT), Canada, the ongoing reliance on country foods (e.g., locally-harvested land mammals, fish, birds and plants) may influence people’s exposure to environmental contaminants, potentially causing significant differences in exposure profiles relative to the general population of Canada. Country food consumption in First Nations peoples has been associated with improved nutrition, food security, and lower rates of chronic disease [1–3]; however, such foods can also pose potential risks via exposure to contaminants such as mercury and cadmium. Human biomonitoring is a validated method used in studies to quantify environmental exposure to both natural and anthropogenic chemicals and contaminants. For example, a human biomonitoring approach is employed within the Canadian Health Measures Survey (CHMS) to characterize profiles of exposure in the Canadian population [4–7]. Due likely to logistical issues such as low density population in the region, and expense, neither the Health Canadian Measures Survey nor the First Nation Biomonitoring Initiative sampled communities of the Northwest Territories. To address these concerns, our team has been conducting an ongoing multi-year contaminant biomonitoring study to investigate current levels of contaminant exposure among participating Dene First Nations communities of the Canadian subarctic.

This paper describes the implementation of this contaminant biomonitoring project, which included three types of biological sampling (human hair, urine, and blood), as well as the administration of three questionnaires. The questionnaires included a risk perception and messaging survey (i.e., the Health Messages Survey), a food frequency questionnaire (FFQ), and a 24-h dietary recall survey. The aim of the project is to provide baseline reference levels for this region, to be monitored over time, and to assess the risk and benefits of country foods consumption for contaminant and nutrient exposure. The current work reports the methodology of the implementation of the biomonitoring project and key findings of the Dehcho region of the six communities that accepted to participate in the biomonitoring, including: Deh Gah Gotie; West Point; Jean Marie River; Ka’a’gee Tu; Sambaa Ke, Katlodeeche. As part of the larger biomonitoring project, the research in the Sahtú region is still ongoing and results should be available in 2019.

## Methods

A community-based project was designed [8] based on consultations that began in 2014.

### Participant inclusion and consent

#### Participation criteria

Community members aged six years and older were eligible to participate, regardless of sex, family status, or ethnicity. Exclusion criteria included young children, individuals who were unable to provide free and informed consent (e.g., those under the influence of drugs or alcohol, individuals with Alzheimer’s disease, as well as any minors who were unable to obtain the consent of their parent or guardian), and individuals unwilling to receive their personal results.

#### Participant recruitment

In total, 6 communities of the Dehcho region have participated in the project. In each community details about the project were disseminated through posters placed in public spaces throughout the community, local radio interviews and media appearances. The researchers sampled proportional to community population size, with a minimum target of 10% of the population per community. Overall, it was aimed for participants to represent the sex and age distribution of the combined population (i.e., children, young adults, elders); recruitment efforts were designed to ensure this representation.

In communities with a population of 100 or less, hereafter referred to as small communities, all community members were invited to participate. Participants were recruited by the local coordinator, who delivered posters to all residents’ mailboxes, and called all residential telephone numbers from a list which was provided by the band office directly to the local coordinator from the community who was hired to assist the project. Up to two telephone calls were made in the week prior to conducting the contaminant biomonitoring study, and the posters were delivered about one week before the clinic. The coordinator invited the household to participate, and described the aim of the study, details of remuneration, the confidential nature of the study, and the dates and location where the study was to be conducted.

In communities with a population over 100, hereafter referred to as large communities, participants were included in two ways. To respect the will of the community partners, any community member who volunteered to participate was included. Firstly, participants were recruited by contacting a random sample of households and inviting all members of these households to participate. When up-to-date and comprehensive community household lists were unavailable (e.g., no detailed maps with explicit addresses; no exhaustive lists; no sufficient Google maps data), this information was compiled from partial lists of landline phone numbers. From these lists, telephone numbers were randomly sampled using a random number generator.

The number of households contacted in each large community was based on the number of individuals in each household, the community’s population (from e.g., Government of Northwest Territories’ Statistics department [9]), and the total number of landlines within the community. To obtain at least 10% of the residents of the community, calculation assumed that 25% of contacted households would participate. As such, 40% of the residents were randomly selected and were contacted. By example, in a community of 1000 residents with 200 phone numbers on the list, 80 numbers were randomly selected to reach potentially 400 individuals (5 by household).

The local coordinator telephoned households up to two times in the week prior to the biomonitoring sampling clinic study. If the coordinator could not reach a household member or could not leave a voicemail, the next randomly-selected telephone number was called. When a household member was reached, the coordinator followed the same process described above for small communities. If any disparities in participant recruitment were noted, in terms of certain demographics (i.e., children/young adults/elders/male/female) being underrepresented in the sample, additional recruitment efforts were made to invite these groups to take part in the study. At the end of each clinic day, researchers verified the age and sex distribution of the sample to-date, and the local coordinator was invited to do targeted recruitment of any under-represented groups by phone calls. Researchers visited local schools to introduce the project to teachers and students when possible, to facilitate recruitment of children. These steps helped promote the representativeness and generalizability of results.

#### Consent forms

The research team described the project to individuals who came to the biomonitoring clinic. Participants were required to confirm their understanding of the project by providing written consent and signing the consent form. For those under 18 years of age, the research team described the project both to the minor and to their parent/legal guardian. The minor thereafter was required to confirm his/her understanding of the project and provide verbal assent, while the parent/guardian of the child was required to provide written consent by signing the consent form. Translated (North Slavey) consent documents were also available upon request. Questionnaires were written and administered in English; therefore, local Indigenous language experts were hired to assist participants in translation as necessary.

Free consent, and participant confidentiality and anonymity were among several measures taken to ensure the ethical integrity of the study. Participants were invited to opt-in to any or all of the six project components (i.e., the three questionnaires and three biological samples).

### Research and ethics licences

In preparation for the project, the research team received annual ethics clearance for the project design and methods. Ethics and licensing reviews were conducted by the University of Waterloo Research Ethics Committee, the Stanton Territorial Health Authority (STHA) for Human Research, and the Aurora Research Institute. Health Canada ethics approval was also obtained regarding additional analysis of the biobanked samples.

### Biobank

Within the consent form, participants could opt to have their samples stored in a biological sample bank (i.e., biobank). To this end, aliquots of participants’ samples were stored for up to 10 years so that contaminant and nutrient biomarkers not included within the original suite of analytes examined can be quantified in the future. As per each Community Research Agreement, no genetic or drug testing will be conducted on biobanked samples. Any new findings will be reported to participants and the community as this information becomes available.

### Participant compensation

Depending on how many components of the project a participant opted into, they could spend up to two hours in the biomonitoring clinic. In recognition of this time commitment, each participant received a $25 gift card to a local general store (Northmart/Northern/Ehdah Cho Store). Furthermore, each participant was able to enter their name into a draw for the chance to win a $250 gift card to any Northmart Food and Retail Store.

### Biomonitoring implementation

Human biomonitoring investigations serve as a tool to measure the cumulative chemical exposure at the human level, reflecting the contributing effects of the immediate environment [10–13]. This methodology is employed by national organizations worldwide to monitor the health indicators of a population [5, 14, 15] and is time- and space-dependent [16, 17]. Biomonitoring data contribute to a greater understanding of exposure to environmental contaminants and serve to inform effective risk assessment and management.

### Preparation

In each of the project years (2016–2018), sample and data collection was completed during the winter months (November to March), to reduce the potential impact of seasonal variability in the results reported. Winter sampling facilitated travel between communities; accommodations for the research team and venues for the biomonitoring clinic were more available; low temperatures and seasonal harvest patterns helped ensure that the majority of residents were in participating communities at the time of the study. However, winter sampling also introduced several challenges. For example, in some cases, the opening of winter roads can sometimes increase travel among communities and is a popular time to travel out of town to visit friends and family. Furthermore, early winter (thin ice) and the mid-winter (coldest) are also periods when the consumption rates of fish (e.g. whitefish, lake trout) and game (e.g. beaver, rabbit) are often lower than observed during other parts of the year.

Between two to five research team members from the University of Waterloo traveled to each participating community and met with the local coordinators and the nurse for the collection of samples and data. Prior to the research team’s arrival, all the required sampling and survey equipment were shipped to, and stored in, secure storage facilities within the two project hubs (i.e., Hay River, and Yellowknife, NT). From these hubs, project supplies and equipment were packed in Pelican Cases and Action Packers and shipped to each participating community. The research team coordinated with local band offices to rent space in local community centres for anywhere from two to eight days, to establish the biomonitoring clinic.

### Data collection forms

After completing the informed consent process, participants were asked to provide basic demographic and personal information (e.g., age, sex, smoking status). In addition, a member of the research team recorded responses to additional questions (e.g., food consumption in the previous two hours, alcohol and smoking use, medications). A confidential participant identification number (ID) was used in lieu of any potentially identifying information on all forms and questionnaires.

### Anthropometry

For anthropometric measures, participants were invited to a station where the research team measured the individual’s weight in kilograms on a medical balance (Health o meter® 753KL Legal for Trade Medical Scale, McCook, IL, USA). The participant’s height was also measured in centimeters by using a standard tape measure affixed to a nearby wall.

### The 24 Hour recall survey

Participants were invited to complete two dietary surveys. For the first survey, participants were asked to detail what they had eaten over the previous 24 h using a local adaptation of the web-based survey of dietary patterns and behavior developed by Hanning and colleagues (WEB-Q, https://www.peaceworks.ca/web-q) [18]. This survey has been validated [19] and used to obtain food group and nutrient intakes among youth in the coastal Mushkegowuk First Nations in Ontario, Canada [20]. The survey uses a multi-pass technique to sequentially question participants about foods consumed according to meal occasion, food details (e.g., methods of preparation, portion sizes selected from six options and associated photographic images), and commonly missed additions to foods (e.g., condiments, cream added to coffee) to simulate a 24 h dietary recall interview [21]. The survey includes a bank of approximately 900 food and beverage options. For this research, local traditional country foods and locally-obtained photos of these foods, as prepared, were added to assist food selection and portion size estimation. The survey was also adapted to include functional foods with enhanced fatty acid content (e.g., omega-3 eggs) which are available within grocery stores in larger communities. Generally, individuals with grade six literacy are able to complete the computer-based survey on their own; however, the members of the research team were available to assist participants with the survey as needed. After completion of the survey, all responses were transferred for nutrient intake analysis based on the most recent Canadian Nutrient File data [22]. The 24 h recall survey was also used to generate the portion size information necessary to estimate nutrient and contaminant intakes from the second dietary survey, a food frequency questionnaire (FFQ). As a web-based survey, the main limitation of the WEB-Q was the availability of Wi-Fi access. To remediate to this issue, when Wi-Fi was not otherwise available in the biomonitoring clinic, Wi-Fi hotspots were created by the research team using a ZTE mobile turbo-hub over the cellular network.

### Food frequency questionnaire (FFQ)

This survey was based on the questionnaire previously implemented by researchers from the Centre for Indigenous Peoples’ Nutrition and Environment (CINE) in the Northwest Territories in the 1990s [23, 24] and validated for the project in 2015. The FFQ gathered information about the country food consumption patterns of study participants over the previous year. The previously paper-based survey was adapted into an e-survey administered on iPads through the QuickTapSurvey app (www.quicktapsurvey.com). Survey questions asked about the types of country foods consumed, the frequency by which these foods were consumed, and the various methods used to prepare the food, all of which are factors that can influence dietary contaminant exposure. For example, food preparation methods can impact the in vitro bioaccessibility of mercury from consumed fish [25–27]. A range of preparation methods were documented (cooked, pan fried/deep fried, grilled/roasted/baked, smoked, raw, boiled/soup/stew, smoked/fully dried, smoked/half dried, campfire). As with the 24 h recall survey, participants were generally able to complete the questionnaire on the iPads on their own. However, a member of the research team was available to assist participants with the completion of the survey when needed.

### Health messages survey

Participants were invited to respond to a short survey on contaminants, including questions that evaluated: i) awareness and understanding of current health messages on country foods and contaminants; ii) perception of risks related to contaminant exposure; iii) perspectives on human health and country foods; and iv) preferences for receiving health and contaminant information. The survey was administered using the same iPad app used for the FFQ. Questions about participants’ awareness of health advisories for mercury and cadmium in fish and moose meat respectively, were made specific to their regions. Risk perception questions were adapted from those previously used in Nunavut and Nunavik, Canada [28]. The survey was reviewed and tested in an open forum prior to its introduction into the contaminants biomonitoring study. This forum, which happened during a camp on the land, showed the importance to adapt the survey to local culture. By example, the four seasons were not meaningful for participants, and the level of language and terms used was modified (e.g. the term traditional foods was preferred over country foods). Due to the level of knowledge assessed, participants under 12 years old were not invited to complete the health messages survey.

### Biological samples

Hair, blood, and/or urine samples were collected from each participant who agreed to provide biological samples for chemical analysis. The participant ID number affixed to the data collection sheet was recorded on each biological sample. The time of day of collection for blood and urine was recorded, along with the time of the previous urine voiding, and confirmation of a provided hair sample was recorded. In order to protect the privacy of participants, no other potentially-identifiable information was recorded with or on the sample.

### Blood samples

For those participants who agreed to provide a blood sample, a registered nurse drew blood from the median antecubital vein of the anterior forearm with a 21G or 23G collection set (BD Eclipse, Becton Dickinson, Rutherford, NJ). Blood samples were collected in one metal-free plastic 6 mL-vacutainer green tube containing sodium heparin (BD Vacutainer™, Becton Dickinson, Rutherford, NJ) for whole blood metals analysis and 2 plastic 6 mL-vacutainer lavender tubes containing K_2_EDTA (BD Vacutainer™, Becton Dickinson, Rutherford, NJ) for plasma analysis. Once collected, blood tubes were kept at 4 °C in portable coolers (Koolatron, Brantford, ON). Tubes containing whole blood were kept at 4 °C until shipment for metals analysis. The two tubes of blood containing EDTA were centrifuged for 15 min at 3000 rpm in a portable centrifuge (VWR®, Mississauga, ON), separated in 1.2 ml polypropylene vials (Thermo Scientific™ Nalgene™, Waltham, MA) with disposable graduated transfer polyethylene pipettes (Fisherbrand™, Ottawa, ON) and stored at − 20 °C in portable freezers (Whynter, Santa Fe Springs, CA). A plasma vial kept at 4 °C, pre-loaded with dibutylhydroxytoluene (BHT), was sent for fatty acids analysis and a plasma vial stored at − 20 °C was sent for persistent organic pollutants analysis. All remaining vials were kept at − 20 °C until stored at − 80 °C in the biobank for potential future further analyses.

### Urine samples

For those participants who agreed to provide a urine sample, a research team member provided the participant a container on site. Random spot urine samples were collected using a sterile polypropylene 120 ml-container (Fisherbrand™, Ottawa, ON) and separated into 5 × 10 mL aliquots in 15 mL polypropylene tubes (Thermo Scientific™ Nunc™, Waltham, MA). Once aliquoted, two tubes (i.e., 20 ml) of urine were kept at 4 °C until they underwent metal analyses, while all additional tubes were kept at − 20 °C until stored at − 80 °C in the biobank for further analyses.

### Hair samples

For those participants who agreed to provide a hair sample, a small lock of hair was collected by a member of the research team using sterilized scissors, according to procedures (e.g. http://www.fnfnes.ca/gallery/videos) previously established for the First Nations Food, Nutrition and Environment Study (FNFNES) project [29]. Longer hair (about 40 strands) was cut immediately adjacent to the scalp, placed in a polyethylene bag and stapled twice on the proximal side of the lock of hair. For individuals with short hair, a sheet of paper was used to collect hair trimmings, which were evenly gathered from the back of the head, then folded, placed in a polyethylene bag and stapled to ensure that the hairs were secure.

### Shipping

In fly-in communities, lab materials, equipment, and samples (with ice packs in coolers) were shipped via local air carriers (e.g., First Air Cargo, Buffalo Air Express, Northwright Air). This process facilitated the delivery of samples to affiliated laboratories within 72 h. For road-access communities, portable electric freezers with ice packs were used to keep samples frozen prior to shipment from the research hub (e.g., Hay River, NT) using a national courier (e.g., Purolator) within 48 h.

### Laboratory analysis

As per the Northern Contaminants Program (NCP) mandate, chemicals analyzed within biological samples included several metals (e.g., mercury, lead, cadmium) and nutrient biomarkers (e.g., selenium, n-3 polyunsaturated fatty acids (PUFA)) associated with country foods, and persistent organic pollutants (e.g., PCBs, organochlorine pesticides) that undergo long-range transport [30, 31]. However, community consultations indicated a set of contaminant concerns much broader than those that fall under the NCP mandate. According to this input, a broader set of analytes were measured within samples with additional funding from the Population Biomonitoring Section of Health Canada (Table 1). Collectively, these approaches facilitated comparisons to other biomonitoring programs undertaken in Canada, including the International Polar Year Inuit Health Survey (IHS), the Nunavik Inuit Health Survey (NIHS), the First Nations Food Nutrition and Environment Study (FNFNES), the Nituuchischaayihtitaau Aschii Multi-Community Environment-and-Health Study, the FNBI and the CHMS [7, 29, 31–37].

**Table 1:** Laboratory analyses completed during the Human Biomonitoring in the Dehcho Region of the Northwest Territories (2016–2017)

Class	Matrix	Parent compounds	Biomarkers
Metal	Hair	Mercury	Total Mercury
Metal	Urine and whole blood	Aluminium	Total Aluminium
		Arsenic	Total Arsenic
		Barium	Total Barium
		Beryllium	Total Beryllium
		Cadmium	Total Cadmium
		Cesium^a^	Total Cesium
		Chromium	Total Chromium
		Cobalt	Total Cobalt
		Copper	Total Copper
		Gallium	Total Gallium
		Iron^2^	Total Iron
		Lead	Total Lead
		Lithium	Total Lithium
		Manganese	Total Manganese
		Mercury	Total Mercury
		Nickel	Total Nickel
		Rubidium^a^	Total Rubidium
		Selenium	Total Selenium
		Strontium	Total Strontium
		Thallium	Total Thallium
		Uranium	Total Uranium
		Vanadium^b^	Total Vanadium
		Zinc	Total Zinc
Lipids	Plasma	Fatty acids	Omega-3 (EPA + DHA) PUFAs N-3, N-6
POPs- Electrical and coolant fluids	Plasma	Polychlorinated biphenyl (PCB)	PCB, Aroclor 1260
			PCB, IUPAC # 28
			PCB, IUPAC # 52
			PCB, IUPAC # 66
			PCB, IUPAC # 74
			PCB, IUPAC # 99
			PCB, IUPAC # 101
			PCB, IUPAC # 105
			PCB, IUPAC # 118
			PCB, IUPAC # 128
			PCB, IUPAC # 138
			PCB, IUPAC # 146
			PCB, IUPAC # 153
			PCB, IUPAC # 156
			PCB, IUPAC # 163
			PCB, IUPAC # 167
			PCB, IUPAC # 170
			PCB, IUPAC # 178
			PCB, IUPAC # 180
			PCB, IUPAC # 183
			PCB, IUPAC # 187
			PCB, IUPAC # 194
			PCB, IUPAC # 201
			PCB, IUPAC # 203
			PCB, IUPAC # 206
POPs- Flame retardants	Plasma	Polybrominated diphenyl ethers or PBDEs	PBDE, IUPAC # 15
			PBDE, IUPAC # 17
			PBDE, IUPAC # 25
			PBDE, IUPAC # 28
			PBDE, IUPAC # 33
			PBDE, IUPAC # 47
			PBDE, IUPAC # 99
			PBDE, IUPAC # 100
			PBDE, IUPAC # 153
	Plasma	Polybrominated Biphenyls (PBBs)	PBB, IUPAC # 153
POPs- Pesticides	Plasma	Aldrin	Aldrin
		Mirex	Mirex
		Hexachlorobenzene	Hexachlorobenzene
		Chlordane	gamma-Chlordane
			alpha-Chlordane
			cis-Nonachlor
			trans-Nonachlor
			Oxychlordane
		Lindane	gamma-HCH
			beta-HCH
		DDT	p,p’-DDE
			p,p’-DDT
		Toxaphene	Parlar no. 26
			Parlar no. 50

^a^Only available in some blood samples (*n* = 10)

^b^Only available in urine

### Hair mercury analysis

Following shipment, hair samples underwent analysis for total mercury in the laboratory of Dr. Brian Branfireun (Biotron Analytical Services, University of Western Ontario). In this analysis, the 2 cm of hair (30 mg minimum weight) most proximal to the scalp was used. Analyses were completed with a Milestone Direct Mercury Analyzer (DMA-80; Milestone, Sorisole, Italy) using thermal decomposition followed by atomic absorption spectroscopy, as outlined in U.S. Environmental Protection Agency (US. EPA) method 7473 [38]. To validate the batch analysis, a standard reference material (IAEA-086) was run at the beginning and end of every batch of 20 samples, with no less than five blanks run in each sample batch. Certified reference materials were used to validate the method and percent recoveries were determined using: National Institute of Standards and Technology (NIST) 1566B, oyster tissue, NIST 2976, mussel tissue, NIST 2974a, freeze dried mussel tissue; National Research Council of Canada (NRC) DORM-3, fish protein; National Institute of Environmental Studies (NIES) No. 13, human hair; and International Atomic Energy Agency (IAEA) 086, human hair. A single sample was run in triplicate in each sample batch and the mean relative standard deviation of the triplicates measured to ascertain measurement precision. The method detection limit was determined as three times the standard deviation of the blanks. The limit of detection was 0.05 ng. The recovery for all controls exceeded 80% and the reproducibility variation fell below 20%.

### Metal analyses in blood and urine

Metals in whole blood and urine samples were analyzed at the Université de Montréal using an inductively coupled plasma mass spectrometer (ICP-MS) in the laboratory of Michèle Bouchard in the Department of Environmental and Occupational Health. In total, 24 metals were quantified in both urine and blood. After sample preparation, metals were brought to their elementary form by passing through an argon plasma for quantification by an Agilent 7700 Series Inductively Coupled Plasma Mass Spectrometer (ICP-MS). All the samples were run in triplicate. Reference materials (ClinChek urine control 8847–8849 lot: 122; ClinChek urine control 8847–8849 lot: 122; INSPQ blood control QM-B-Q1409; INSPQ blood control QM-B-Q1505) were used to assess the method’s quality; recovery exceeded 90%. The limit of detection (LOD) in blood and urine were between 0.01 (e.g. arsenic in blood; uranium in urine) and 4 μg/L (selenium in blood). In addition, the laboratory is enrolled in the NCP interlaboratory study. Urinary mercury analysis was conducted at the Centre de Toxicology du Québec (CTQ) using a single-quadrupole PerkinElmer ICP-MS system (Shelton, USA) with a method having a limit of detection of 0.46 μg/L. The reproducibility variation was 12%.

### POPs analysis

Analysis of persistent organic pollutants (POPs) in plasma were conducted in the laboratory of the Centre de Toxicologie du Québec (CTQ), as previously described [39]. POPs were extracted by liquid extraction before being analyzed by an Agilent gas chromatograph (GC) coupled to a mass spectrometer (Agilent Technologies; Mississauga, ON). Reference materials (e.g. National Institute of Standards and Technology SRM-1958) were used to assess the method’s quality; and recovery exceeded 90%. The LODs were between 0.005 (e.g. PCB128, PCB153) and 0.5 μg/L (PCB128). Recoveries varied from 68 to 90%. The reproducibility variation was below 13%.

### Fatty acid analysis

The fatty acid composition of plasma was determined by high throughput gas chromatography by Dr. Ken Stark in the Department of Kinesiology at the University of Waterloo [40]. Briefly, lipids were extracted from the samples using 2:1 chloroform:methanol, and were transesterified to fatty acid methyl esters using 14% boron trifluoride in methanol in the presence of an internal standard (22:3n-3 ethyl ester; Nu-Check Prep, Elysian, MN, USA) to allow for quantification. The fatty acid methyl esters were analyzed using fast gas chromatography with flame ionization detection (Varian 3900) equipped with a DB-FFAP capillary column (Agilent Technologies). Fatty acid peaks were identified by retention time matching to a mixed reference standard (GLC-462, Nu Chek Prep, Inc.) and gas chromatography coupled to mass spectrometry was used to confirm the identify of any unknown peaks.

### Biobanked samples

Additional analytes (i.e., those that fall outside of the NCP mandate) were measured within biobanked samples provided by participants. To date, these biomarkers have included phthalates, cotinine, polycyclic aromatic hydrocarbons (PAHs) and arsenic species (Table 2). Phthalates can be found in certain types of food packaging and, therefore, communities with limited access to fresh foods may face elevated levels of these contaminants [41]. Exposure markers of inorganic arsenic (e.g., arsenate, arsenite, and methylated arsenic metabolites) are known human carcinogens produced in large quantities in historic gold mines in the Northwest Territories [42]. PAH exposure may also be elevated among Indigenous communities in the Northwest Territories due to frequent, severe forest fires in the region [43]. Lastly, because smoking can contribute substantially to cadmium exposure, cotinine, the predominant metabolite of nicotine was also measured, yielding information regarding potential sources of cadmium exposure among study participants. These analyses were conducted in the laboratory of the Centre de Toxicologie du Québec (CTQ) [44, 45]. Finally, hair metals were analysed at University of Montreal using the ICP-MS method described above. Altogether, these analyses provide a more complete understanding of contaminant exposure in First Nations communities of the Northwest Territories.

**Table 2:** Supplemental analyses completed in biobanked urine samples of the Human Biomonitoring in the Dehcho Region of the Northwest Territories (2016–2017)

Class	Parent compound	Biomarkers
Polycyclic aromatic hydrocarbons (PAHs)	Anthracene	1-Hydroxybenz(a)anthracene
		3-Hydroxybenz(a)anthracene
	Benzo(a)pyrene	3-Hydroxybenzo(a)pyrene
	Chrysene	2-Hydroxychrysene
		3-Hydroxychrysene
		4-Hydroxychrysene
		6-Hydroxychrysene
	Fluoranthene	3-Hydroxyfluoranthene
	Fluorene	2-Hydroxyfluorene
		3-Hydroxyfluorene
		9-Hydroxyfluorene
	Naphthalene	1-Naphtol
		2-Naphtol
	Phenanthrene	1-Hydroxyphenanthrene
		2-Hydroxyphenanthrene
		3-Hydroxyphenanthrene
		4-Hydroxyphenanthrene
		9-Hydroxyphenanthrene
	Pyrene	1-Hydroxypyrene
Cotinine	Cotinine	Free Cotinine
Phthalates	Benzyl butyl phthalate	Mono-benzyl phthalate (MBzP)
	Dicyclohexyl phthalate	Mono-cyclohexyl phthalate (MCHP)
	Di-n-octyl phthalate	Mono-3-carboxypropyl phthalate (MCPP)
		Mono-n*-*octyl phthalate (MnOP/MOP)
	Diethyl phthalate	Mono-ethyl phthalate (MEP)
	Di-isobutyl phthalate	Mono-isobutyl phthalate (MiBP)
	Di-isononyl phthalate	Mono-isononyl phthalate (MiNP)
	Dimethyl phthalate	mono-methyl phthalate (MMP)
	Di-n-butyl phthalate	Mono-n-butyl phthalate (MnBP)
	Di-2-ethylhexyl phthalate	Mono-(2-ethyl-5-hydroxyhexyl) phthalate (MEHHP)
		Mono-2-ethylhexyl phthalate (MEHP)
		Mono-(2-ethyl-5-oxyhexyl) phthalate (MEOHP)
		*Mono-*(*2-ethyl-5-carboxypentyl) phthalate* (MECPP)
Arsenic species	Arsenic	Dimethylarsinic acid (cacodylic acid)
		Monomethylarsonic acid
		Inorganic arsenic
		Arsenocholine + Arsenobetaine
		AsIII (arsenite)
		AsV (arsenate)

### Interpretation of biomarker results

For the majority of contaminants defined in Tables 1-2, no defined health-based tissue guidance values were available. The main exceptions to this included mercury, cadmium, and lead; the Health-Based Tissue Guidance (HBTG) values for these metals are reported in Table 3. These values (Table 3) were based on those previously used as thresholds indicating regular or early notification follow-up with participants [4, 34, 46–52].

**Table 3:** Follow up Health-Based Tissue Guidance (HBTG) values used during the Human Biomonitoring in the Dehcho Region of the Northwest Territories (2016–2017)

Contaminant	Sample	Regular Follow-up	Early Notification
Mercury	Hair	> 5 μg/g^a^ (2 μg/g)^a^	> 25 μg/g^a^ (10 μg/g)^a^
Mercury	Blood	> 20 μg/L^c^ (8 μg/L)^c, e^	> 100 μg/L^c, d, e^ (40 μg/L)^c^
Mercury	Urine	> 25 μg/L^h^	
Cadmium	Blood	> 5 μg/L^b, d, f^	
Cadmium	Urine	> 7.3 μg/L^b^	
Lead	Blood	> 100 μg/L^d, g^ (50 μg/L)	> 200 μg/L^b, d, g^ (100 μg/L)^b, d^
Lead	Urine	> 7 μg/L^b^	
Uranium	Urine		> 15 μg/L^i^

The values are identified according to the vulnerability level of individuals to contaminants: for the group considered as least vulnerable such as men over the age of 17 and women over the age of 45, as well as for the most vulnerable group, including minors and women of childbearing age(males 6–17 years of age and females 6–45 years of age)

^a^Delormier, T, 2012. Synopsis of Research Conducted Under the 2010–2011 Northern Contaminants Program

^b^Haines, D.A., et al., 2011. Reporting results of human biomonitoring of environmental chemicals to study participants: a comparison of approaches followed in two Canadian studies

^c^Legrand, M., et al., 2010. Methylmercury Blood Guidance Values for Canada

^d^Nieboer E, et al., 2013. Nituuchischaayihtitaau Aschii Multi-community Environment-and-Health Study in Eeyou Istchee 2005–2009

^e^Smith, L., et al., 2009. Maternal-Infant Research on Environmental Chemicals (MIREC)- Managing Maternal Blood Mercury Levels

^f^Smith, L., et al., 2008. Maternal-Infant Research on Environmental Chemicals (MIREC)- Managing Maternal Blood Cadmium Levels

^g^Smith, L., et al., 2008. Maternal-Infant Research on Environmental Chemicals (MIREC)- Managing Maternal Blood Lead Levels

^h^Schulz et al., 2011. Update of the reference and HBM values derived by the German Human Biomonitoring Commission

^i^Agency for Toxic Substances and Disease Registry, 2009. Case Studies in Environmental Medicine (CSEM) - Uranium Toxicity

For contaminants without established health-based guidance values, the research team compared the participants’ levels of exposure with those normally observed in nationally-representative Canadian biomonitoring studies (e.g., CHMS, FNBI) [5–7, 31]. The upper limit of the normal range of exposure for these analytes were defined according to age- and sex-specific 95th percentiles established from the CHMS and FNBI. Beside the use of the guidance values, re-testing was also assessed on a case by case basis (e.g. arsenic in urine) but no participant from the Dehcho had levels requiring a second sample to complete a follow up re-testing.

A customized report generation software, created in Microsoft Excel™, was used to compare participants’ contaminant exposure profile to each of the available health-based tissue guidance values and 95th percentile values from the CHMS and FNBI. The follow-up process for participants’ results is summarized in Additional file 1: Fig. S1. When levels of cadmium, mercury, or lead exceeded one of the regular follow-up guidance values, participants were offered re-testing while the research team returned for the community results presentation within 12 months of initial sample collection. This follow-up mechanism also provided these participants with the option of inviting their household members to provide follow-up samples as well. When participant levels’ exceeded one of the early-notification guidance values, the participant would receive a phone call from a member of the research team as soon as possible. Through these follow-up procedures, the research team provided information on how individuals could reduce their exposure.

The offering of follow-up testing to participants with biomarker levels exceeding health-based guidance values helped assess whether exposures had remained elevated since the initial sample collection. Participants who presented profiles of contaminant exposure that exceeded early notification guidance values were referred to a physician for medical examination. Three urine spot samples were recommended in the follow up period (3 months), which was determined to be the minimum required number of random urine voids to be representative of metals (e.g., cadmium) exposure [53]. Depending on which contaminants presented elevated levels, the re-test procedure occasionally indicated the collection of blood samples rather than urine.

## Results

The results presented here are from the Dehcho region. The multi-year project is ongoing in the Sahtú Region.

### Characteristics of participants

The participants came from six First Nations communities from the Dehcho Region. A total of 283 participants were recruited, representing 8.3% of the residents from this region (*n* = 3473) and 20.6% of these communities (12–40% for each participating community). However, four participants chose to withdraw from the study, their information was thereafter removed and their results are thereby not included in the results reported herein. Participant included 51.3% female and their age was distributed uniformly with a median of 42, a mean of 39.1 and a range from 6 to 79 years old. A total of 52 minors participated in the project. The age and sex distribution within all the Dehcho communities were estimated by the Bureau of Statistics of the Northwest Territories [53]. Noting that no participants were recruited under the age of 6, the age categories distribution in the Dehcho for 10–14 (6.9%), 15–24 (13.7%), 25–44 (29.4%), 45–59 (22.6%) and 60+ (16.0%), is similar to the current sample, respectively of 9.8, 17.3, 24.4, 28.2 and 16.9%. Characteristics of participants, including smoking and alcohol consumption in the last 24 h, as well as body mass index are presented in Table 4. As people completed any component they wanted, the overall number of participants differ between these components, with the most number of participants completing the FFQ survey and providing a hair sample, and the least number of participants completing the Health Messages Survey and providing a urine sample.

**Table 4:** Characteristics of participants from the Dehcho Region (*n* = 279)^a^ of the Human Biomonitoring (2016–2017)

Parameters	Values
Age	Range: 6 to 79 years old
	Mean: 39.1 years old
	Refusal/ND: 4.7%
Sex	Males: 48.7%
	Females: 51.3%
	Refusal/ND: 0%
Smoking status (in the last 24 h)	Smokers: 32.3%
	Non-smokers: 67.0%
	Refusal/ND: 0.7%
Alcohol consumption (in the last 24 h)	Alcohol: 9.7%
	No alcohol: 86.7%
	Refusal/ND: 3.6%
Body mass index - for adults only (+ 18)	Range: 17.7 to 60.0
	Mean: 28.7
	Refusal/ND: 16%

^a^These values do not include the 4 participants who chose to withdraw from the study

### Dietary behavior of country food consumption

A total of 156 participants completed the FFQ. The 10 most eaten country foods (and the percent of participants who reported eating each of those foods) are reported in Table 5. Moose (92%), whitefish (89%) and rabbit (57%) were consumed by the largest number of participants. The Mackenzie River and Great Slave Lake were the main waterbodies from which fish were obtained. Although the majority of participants reported eating fish only from waterbodies that did not have a Government of the Northwest Territories consumption notice [54], 33% of participants reported eating fish from a specific waterbody that had a consumption notice for mercury. The medicinal plants consumption was noticeable as 37% respondents reported consuming at least one medicinal plant or herbal preparation within the previous year (e.g., Labrador tea, rat root, spruce gum).

**Table 5:** The country food the most consumed and the average frequency by week over one year within the respondents from the Dehcho region of the Human Biomonitoring (2016–2017)

	Country Food Consumed	Latin name^a^	Percent Consuming (%)	Average Frequency (days/week)
1	Moose	*Alces alces*	92	1.9
2	Whitefish	*Coregonus sp.*	89	1.6
3	Rabbit	*Sylvilagus sp./ Lepus sp.*	57	1.3
4	Northern Pike	*Esox lucius*	57	1.2
5	Canada Goose	*Branta canadensis*	53	1.1
6	Lake Trout	*Salvelinus namaycush*	53	0.9
7	Walleye	*Sander vitreus*	47	1.2
8	Wild Strawberries	*Fragaria sp.*	46	1.1
9	Mallard	*Anas platyrhynchos*	46	1.1
10	Wild Raspberries	*Rubus sp.*	46	1.1

^a^Working Group on General Status of NWT Species. 2006. NWT Species 2006–2010 - General Status Ranks of Wild Species in the Northwest Territories, Department of Environment and Natural Resources, Government of the Northwest Territories, Yellowknife, NT. III pp. http://www.nwtspeciesatrisk.ca/sites/default/files/nwt_species2006.pdf

A total of 132 participants completed the 24-h recall survey. Results showed that, on the day prior to sample/data collection in winter, 5.1% of total Calories consumed in the community were from wild-harvested country foods, with an average for participants of 4.9% (range 0 to 37.6%). Of those participants who completed the survey, 30.3% reported consuming country food in the previous 24 h. Since the research took place in winter (November to February), a majority of participants reported moose meat as the main type of country food consumed, and participants also reported having eaten whitefish, catfish, bass fish, rabbit, caribou, beaver and bison.

### Health messages survey

A total of 44 participants completed the Health Messages Survey. Results showed that 98% of the respondents reported consuming country foods, and that at least 27% would prefer to solely eat country foods (rather than store-bought or a mix of both country and store foods). This result underlines the importance of country foods, and indicates how country foods are favoured. Within other statements, 89% of respondents indicated that they have heard or seen messages saying that *Country foods can provide a significant variety of nutrients*. In addition, 68% of respondents heard a message on mercury in fish and 55% reported since hearing this message being more concerned about the fish they eat. More than half (59%) of respondents reported that they were concerned about the quality and safety of the country foods they consume. Although participants were most concerned with mercury in country foods, they were also concerned about metals (lead, uranium, radon, etc.), and other sources of contaminants such as chlorine in drinking water, indoor air quality, pesticides, and antibiotics in meat.

### Contaminants and nutrients levels

A total of 231 hair, 136 blood and 85 urine samples were collected. For a few participants (*n* = 5), mercury levels in hair and blood were higher than the HBTG values and required a regular follow-up. Two percent of the participants were offered a re-testing for contaminants over the chosen HBTG values. Table 6 provides biological results for the contaminants for which we established thresholds. Only the blood lead and blood cadmium had a geometric mean (GM) above the Canadian Health Measure Survey (cycle 4), but below the First Nations population in Canada (FNBI) [31]. However, the lower limit of the confidence interval are below the CHMS GM. None of the participant’s levels were above the early notification threshold. Within the POPs, only Arochlor 1260 has threshold limit value emitted in the past by Health Canada; however the value was suggested for whole blood while the current value reports plasma levels [55]. Overall, the rates of level above the guidance value were very low and show the relatively low exposure for these contaminants.

**Table 6:** Contaminants levels for which guidance value were established used during the Human Biomonitoring in the Dehcho Region of the Northwest Territories (2016–2017)

Contaminant	Sample	% Detection	GM (IC95)	% Samples over guidance value
Mercury	Hair	> 95%	0.39 μg/g (0.31–0.47)	2.2
Mercury	Blood	40–50%	0.13 μg/L (0.085–0.18)	0.74
Mercury	Urine	50–60%	0.46 μg/L (0.37–0.55) or 0.51 μg/g crea. (0.43–0.62)	0
Cadmium	Blood	> 95%	0.53 μg/L (0.43–0.66)	0
Cadmium	Urine	> 95%	0.31 μg/L (0.26–0.37) or 0.34 μg/g crea. (0.29–0.39)	0
Lead	Blood	> 95%	11 μg/L (9.5–12)	0
Lead	Urine	> 95%	0.50 μg/L (0.41–0.61) or 0.55 μg/g crea. (0.46–0.65)	1.2
Uranium	Urine	> 95%	0.0057 μg/L (0.0047–0.0069) or 0.0062 μg/g creat. (0.0050–0.0077)	0

The metals quantified in urine had a detection rate between 54 and 100%. However, only nine of these had detection rates over 50% in blood (cadmium, copper, lead, manganese, nickel, rubidium, selenium, strontium, zinc). A total of 14 POPs in plasma had a detection rate higher than 50% of the samples. This was the case for: Aroclor 1260, Hexachlorobenzene, p,p’-DDE, trans-Nonachlor, PBDE # 47, PCB # 138, PCB # 153, PCB # 163, PCB # 170, PCB # 180, PCB # 187, PCB # 194, and PCB # 201.

Omega-3 polyunsaturated fatty acid status as assessed by the sum of the percentage of eicosapentaenoic (20:5n-3, EPA) and docosahexaenoic acid (22:6n-3, DHA) in total; fatty acids was 1.8% (between 0.86 and 4.8%), and similar to what has been observed in previous studies in Canadians (1.5–2.4) [56]. Omega-3 fatty acids calculated from the concentration of EPA + DHA in plasma was on average 72 mg/L (between 25 and 189 mg/L).

## Discussion

Country foods provide the people of the Northwest Territories many benefits. These include nutritional, economic, social, and cultural benefits. It is, therefore, important that messages about contaminants and country foods are carefully crafted in a way that balances benefits and risks. The biomonitoring results in the Dehcho show that participants’ heavy metal exposure was generally low (usual range); few participants (2.1%) had samples above the recommended health guideline. However, most of the contaminants measured in this project do not have biological guidelines in biological human samples to distinguish whether the participants’ levels have exceeded those that are estimated to be safe. In fact, 2 % of participants with levels over the guideline requiring a follow up for mercury, and 1 % requiring a follow up for lead. Therefore, although some country foods occasionally have elevated levels of mercury and cadmium, peoples’ exposures in the territory have typically remained low.

Although different levels of toxic metals (mercury, lead and cadmium) were detected among participants, it was the same for some biomarkers for metal nutrients (e.g., selenium and zinc) and omega-3 fatty. High levels of omega-3’s may help protect against heart disease [57] and some country foods (e.g., Lake Whitefish, Cisco) are particularly rich in these important nutrients [58]. In addition, the importance of country foods for the population is clear (i.e., % calorie intake; perception; frequency of consumption). In comparison, a project in Northern Ontario reported that traditional food consumption provided 6% of the total energy intake in winter 2002, more than sixteen years ago [59]. Country foods are generally low in saturated fat and rich in nutrients and significantly contribute to Arctic and sub-arctic dietary quality [24], as such, a shift towards a diet with an increased consumption of store-bought food could negatively impact health. These results reinforce the important message that the benefits of country food consumption generally outweigh contaminant risks.

Sources of contaminants will be investigated in further works. Some are already believed to come from specific food sources. Mercury exposure is known to come mainly from fish consumption [60], while uranium exposure can be from contaminated soil, dust, or drinking water. There are many ways by which people can be exposed to arsenic, including from naturally rich soil or contaminated soil, house dust [60], and harvesting berries from near contaminated sites. Many parts of the Northwest Territories have naturally high levels of arsenic and the Giant Mine near Yellowknife also contribute to release arsenic in the surroundings [61]. In addition, the smoking rate reported is relatively high, increasing the exposure for cadmium and lead. The practice of using lead bullet ammunition and lead fishing jiggers is still ongoing, contributing also to lead exposure. The research team will continue analysing the distribution of the contaminants in the region and will investigate any potential sources of contaminant exposure. POPs can travel very long distances through the atmosphere, and because they last a long time in the environment, can be detected many years after their release. The majority of the POPs listed in Table 8 have not been used or produced in North America for decades. Many of these POPs were banned globally in 2001 through an agreement known as the Stockholm Convention. The levels reported here will enable future studies to monitor the extent to which POP exposures decrease over time as a result of the Stockholm Convention.

Human biomonitoring is relevant to assess aggregated exposures across sources, but alone can rarely detail the relative importance of various exposure sources. With a co-located ongoing project monitoring contaminants in fish in the region, the current study will provide a in-depth view of the exposure through locally caught fish. The characterization from probabilistic models of the profiles for mercury and fatty acids in fish species harvested in this region was a first step to better understand the potential contaminant and nutrients exposure associated with traditional foods [58, 62]. To better assess health risks, the data from the dietary survey could be used to reconstruct individual intake doses integrating the frequency of consumption, the portion size and the preparation method. In addition, a statistical approach is being developed to estimate the relationship between risk factors (e.g. diet, lifestyle, demographics) and the biomarkers levels. The study of smoking status, moose organs consumption and cadmium levels will provide information of the main source of cadmium exposure. The relationship between specific contaminant or nutrition awareness (e.g., mercury in fish) of respondents documented through the Health Messages Survey and the potential impact on fish consumption can also be investigated. In fact, the project was constructed to investigate at multi-levels the relationships between contaminants, nutrition and country foods in a human health perspective.

As the number of participants in this project is limited, the contaminant levels might not be representative of the region. It is a challenge to report precise distributions of exposure of highly variable biomarkers for small populations. It is worth noting that each participant had the choice to provide any type of sample, and complete any survey; even if the characteristics of participants are representative of the group at the community scale, the contaminant levels might not be. Therefore, the results cannot be generalizable to other Dene/Métis communities. Despite lower participation to the surveys than to the biological sampling, the age and sex distribution of the participants who completed these three surveys is similar to the whole group (Females: 43.2–56.3%; average age: 40.1–42.0 years old). The main interest of participants being on the biological results, and the significant time to complete the surveys, are factors believed to have had an impact on the lower rate of completion of the surveys.

Despite a mixed randomized and targeted recruitment, the authors acknowledge other potential bias limiting the representativeness and generalizability of the findings. Selection bias might have been caused by the lack of participation by individuals with fear of having high levels of contaminants, individuals outside of the community (e.g. activities on the land), or individuals with low trust in researchers. Involving the assistance of a research team member to complete the surveys might have increased the desirability bias by increasing the self-report of country food consumption. In addition, imprecision can have occurred though the use of food surveys relying on memory and can also come from the toxicokinetics challenge of using spot samples for contaminants with short half-life in the blood and urine. However, the project fills a gap of knowledge and provides baseline levels in blood, hair, plasma and urine for these community samples.

## Conclusion

The results of this project will report on the links between contaminant exposure, nutritional status, and country food use and will provide important baseline data in this specific region. The outputs of this project will serve to support the development of governance tools, public health interventions, and health policies minimizing the contaminant exposure in Dehcho Indigenous communities of the Northwest Territories.

## Additional file

Additional file 1: **Figure S1.** Organigram of the decision making for biological follow up during the Human Biomonitoring in the Dehcho Region of the Northwest Territories (2016–2017). (JPG 324 kb)

## Abbreviations

BHT: Dibutylhydroxytoluene
CHMS: Canadian Health Measures Survey
CINE: Centre for Indigenous Peoples’ Nutrition and Environment
CTQ: Centre de Toxicology du Québec
DHA: Docosahexaenoic Acid
DHSS: Department of Health and Social Services of the Northwest Territories
DHSSA: Dehcho Health and Social Services Authority
EDTA: Ethylenediaminetetraacetic Acid
EPA: Eicosapentaenoic Acid
FFQ: Food frequency Questionnaire
FNBI: First Nations Biomonitoring Initiative
FNFNES: First Nations Food, Nutrition and Environment Study
GC: Gas Chromatograph
GM: Geometric Mean
HBTG: Health-Based Tissue Guidance Value
HIS: International Polar Year Inuit Health Survey
IAEA: International Atomic Energy Agency
ICP-MS: Inductively Coupled Plasma Mass Spectrometer
LOD: Limit of Detection
NCP: Northern Contaminants Program
NIES: National Institute of Environmental Studies
NIHS: Nunavik Inuit Health Survey
NIST: National Institute of Standards and Technology
NRC: National Research Council of Canada
NT: Northwest Territories
PCB: Polychlorinated Biphenyl
POPs: Persistent Organic Pollutants
PUFA: n-3 polyunsaturated fatty acids
STHA: Stanton Territorial Health Authority
U.S. EPA: U.S. Environmental Protection Agency
