# Supplementary figures and images for: Implementation of human biomonitoring in the Dehcho region of the Northwest Territories, Canada (2016–2017)
Source: Arch Public Health. 2018 Dec 3;76:73. doi: 10.1186/s13690-018-0318-9 (PMC6276191; doi:10.1186/s13690-018-0318-9)

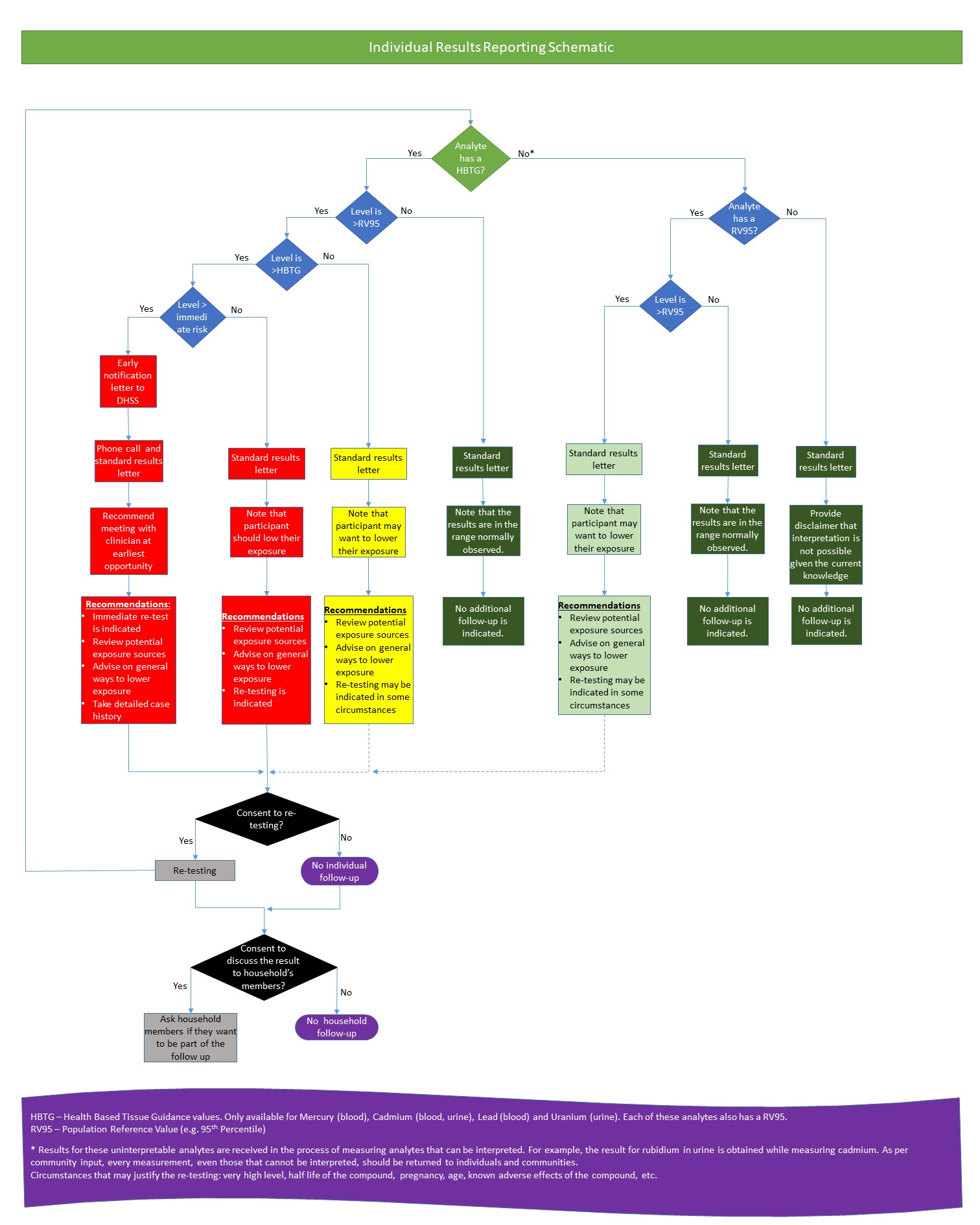

Supplement: Supplementary file 1 — Figure S1. Organigram of the decision making for biological follow up during the Human Biomonitoring in the Dehcho Region of the Northwest Territories (2016–2017). (JPG 324 kb) [file 13690_2018_318_MOESM1_ESM.jpg]
